# Characteristics and risk factors of recurrent pterygium underwent excision combined with limbal conjunctival autograft in southern rural China

**DOI:** 10.3389/fmed.2026.1799127

**Published:** 2026-03-23

**Authors:** Xiaoying Zhu, Meng Fan, Jing Luo, Danyang Liu, Shuaiqing Wang, Ji Hao, Penghua Yuan, Enzhong Jin

**Affiliations:** 1 Department of Ophthalmology, Peking University People's Hospital, Beijing, China; 2 Beijing Key Laboratory of Ocular Disease and Optometry Science, Peking University People’s Hospital, Beijing, China; 3 Department of Ophthalmology, People’s Hospital of Yudu County, Ganzhou, China; 4 Department of Ophthalmology, The First Hospital of Fangshan District, Beijing, China

**Keywords:** pterygium, recurrence, risk factors, rural area, age

## Abstract

**Background:**

Pterygium is a prevalent ocular surface disorder globally. Postoperative recurrence poses a significant challenge to clinical management. Current evidence regarding recurrence risk factors is predominantly derived from urban populations. This study aimed to investigate the specific risk factors for pterygium recurrence within a remote population in Southern China.

**Methods:**

This retrospective, institution-based case series analyzed 200 patients who underwent primary pterygium excision combined with limbal-conjunctival autograft between January 2016 and February 2022. Patients were stratified into recurrent and non-recurrent groups based on postoperative outcomes. Cumulative recurrence rates were estimated using Kaplan–Meier survival analysis. Multivariate binary logistic regression was performed to identify independent risk factors for recurrence.

**Results:**

The mean time to recurrence was 42.21 ± 22.13 months, with a median recurrence-free survival of 72.0 months. Compared to the non-recurrent group, patients in the recurrent group were significantly older (67.62 ± 5.79 vs. 60.27 ± 10.33 years, *p* < 0.001), had lower educational attainment (41.4% vs. 66.1%, *p* = 0.013), reported longer durations of daily sunlight exposure (5.79 ± 1.01 vs. 4.80 ± 2.17 h, *p* < 0.001) and exhibited poorer preoperative best-corrected visual acuity (0.98 ± 0.75 vs. 0.57 ± 0.60 logMAR, p < 0.001). Multivariate analysis confirmed advanced age at onset as the sole risk factor of recurrence (*p* = 0.007).

**Conclusion:**

In rural southern China, pterygium recurrence is distinguished by a delayed onset pattern. Our findings highlight advanced age as a robust independent risk factor in this region, necessitating a shift in clinical focus. Management strategies in underserved areas should prioritize elderly patients through extended follow-up periods and targeted health education to mitigate recurrence risks.

## 1 Introduction

Pterygium is a common ocular surface disorder characterized by subepithelial fibrovascular proliferation, manifesting as a wing-shaped encroachment of tissue extending from the bulbar conjunctiva onto the cornea. This condition presents a substantial public health burden, particularly in China, which exhibited the highest prevalence rate (approximately 53%) in a meta-analysis of 24 countries (1). Given this high prevalence, surgical management is frequent; however, postoperative recurrence remains a persistent clinical challenge. Even with preventive measures such as autologous conjunctival grafting and topical mitomycin C application, reported recurrence rates fluctuate widely—from 0 to 88%—depending on surgical technique and demographic heterogeneity (2–4).

In terms of etiology, previous studies have established well-known risk factors for primary pathogenesis, including ultraviolet radiation, environmental irritants (dust/wind), and viral or genetic determinants (2, 5–7). Regarding postoperative outcomes, factors such as dry eye disease, younger age, and parasite infestation are recognized predictors of recurrence (2, 3, 8). Conversely, the influence of variables such as gender, surgeon experience, and continued sun exposure remains controversial (3, 9, 10).

A critical limitation of the current literature is that the majority of these findings derive from urban settings with robust medical infrastructure. This geographic bias results in a significant underrepresentation of patient populations in China’s remote regions. Residents in these underserved areas face a distinct set of challenges, including harsher environmental exposures, limited healthcare access, and lower health literacy. These unique socioeconomic and environmental stressors suggest that the recurrence risks in remote populations may differ significantly from urban cohorts. Consequently, there is a paucity of research specifically quantifying recurrence rates and identifying risk determinants within these communities.

The present study enrolled a cohort of patients with primary pterygium who underwent surgical excision coupled with limbal conjunctival autografting. We sought to delineate the clinical characteristics and perform a comparative analysis of the risk factors associated with recurrence. By elucidating these clinical profiles, this work aims to refine intervention strategies and inform public health planning, ultimately facilitating the strategic allocation of medical resources in remote or underserved regions.

## 2 Materials and methods

### 2.1 Study design and participants

This retrospective, single-center case series included patients with primary pterygium who underwent excision combined with limbal-conjunctival autografting between January 2016 and February 2022. The study protocol was approved by the Ethics Committee of People’s Hospital of Yudu County and was conducted in strict adherence to the tenets of the Declaration of Helsinki. Written informed consent was obtained from all patients prior to surgery. Inclusion criteria were: (1) residency within the study region; (2) a confirmed diagnosis of pterygium; and (3) a postoperative follow-up period exceeding 12 months. Exclusion criteria included a history of ocular trauma, concurrent ocular pathologies other than cataract, and prior ocular surgeries unrelated to pterygium excision.

### 2.2 Clinical assessment

All patients underwent a comprehensive ophthalmic examination and diagnosis by a designated ophthalmologist (PH. Y.), with confirmation provided by a second senior ophthalmologist (JH. S.). Preoperative assessment included best-corrected visual acuity (BCVA) and slit-lamp biomicroscopy of the anterior segment. Pterygium size was quantified as the length of horizontal encroachment of the fibrovascular tissue from the limbus onto the cornea, measured via slit-lamp examination.

### 2.3 Surgical intervention and data collection

Surgical intervention comprised nasal pterygium excision combined with a superior limbal-conjunctival autograft. Under topical anesthesia, the autograft was subsequently secured to the recipient conjunctival edges and the underlying episclera via interrupted 8–0 Vicryl sutures. Recurrence was defined as the regrowth of any fibrovascular tissue crossing the limbus into the corneal bed at the site of previous excision. Comprehensive medical records were reviewed to document demographic parameters, comorbidities, time to recurrence, clinical characteristics, and laboratory results. Additionally, socioeconomic data, including educational attainment, physical status, and lifestyle factors, were collected. Body mass index (BMI) was calculated based on height (cm) and weight (kg) measurements taken preoperatively. For analysis, educational attainment was dichotomized into lower and higher levels using a cutoff of 6 years.

### 2.4 Statistical analysis

Statistical analyses were performed using SPSS software version 26.0 (SPSS, Inc., Chicago, IL). Descriptive statistics were generated for the demographic and clinical characteristics of the cohort. Cumulative recurrence rates were estimated using the Kaplan–Meier method. Differences in demographic characteristics and perioperative variables between the non-recurrent and recurrent pterygium groups were assessed using independent sample t-tests and chi-square tests, as appropriate. Variables identified as potentially significant in the univariate analysis were subsequently entered into a multivariate binary logistic regression model to identify independent risk factors for recurrence. A *p* value of <0.05 was considered statistically significant.

## 3 Results

The study population comprised 200 patients (50 males and 150 females) with a mean age of 61.34 ± 11.89 years (range, 32–91 years). For the entire cohort, the mean pterygium size was 3.14 ± 0.50 mm (range, 2–8 mm), and the mean preoperative BCVA was 0.63 ± 0.63 logMAR. Bilateral pterygium was observed in only 2 individuals (1.0%) during a mean follow-up period of 30.65 ± 16.96 months (range, 12–83 months). Regarding educational attainment, 125 patients had completed fewer than 6 years of education, while 75 patients had completed more than 6 years. A total of 161 patients reported outdoor occupations, with an average daily sun exposure of 4.94 ± 2.07 h. Comprehensive demographic data, systemic comorbidities, and physical characteristics are summarized in Table 1.

**Table 1:** Demographic and baseline clinical characteristics of investigated pterygium patients.

Characteristics	Patients [*n* = 200]
Male sex, *n* (%)	50 (25)
Average age, years (median, range)	61.34 ± 11.89 (32–91)
Size of pterygium, mm (range)	3.14 ± 0.50 (2–8)
Preoperative BCVA (logMAR)(range)	0.63 ± 0.63 (−0.2–2.6)
Follow-up time (months)	30.65 ± 16.96
Recurrent rate (%)	29/200 (14.5%)
Level of education	
>6 years (%)	125 (62.5)
≤6 years (%)	75 (37.5)
Outdoor Job (%)	161 (80.5)
Sun exposure time, h (range)	4.94 ± 2.07 (0–7)
Smoking (%)	8 (4)
DM (%)	12 (6)
Height (cm)	154.96 ± 8.61
Weight (kg)	56.15 ± 9.56
BMI (kg/m^2^)	23.33 ± 3.15
HDL-C (mmol/L)	1.38 ± 0.31
LDL-C (mmol/L)	3.48 ± 2.48
TG (mmol/L)	1.94 ± 1.64
Total cholesterol (mmol/L)	5.28 ± 1.10

Categorical variables are presented as number (%), whereas continuous variables are reported as mean ± standard deviation. BCVA, best corrected visual acuity; DM, diabetes mellitus; BMI, body mass index; HDL, high-density lipoprotein; LDL, low-density lipoprotein; TG, triglyceride.

**Figure 1:**
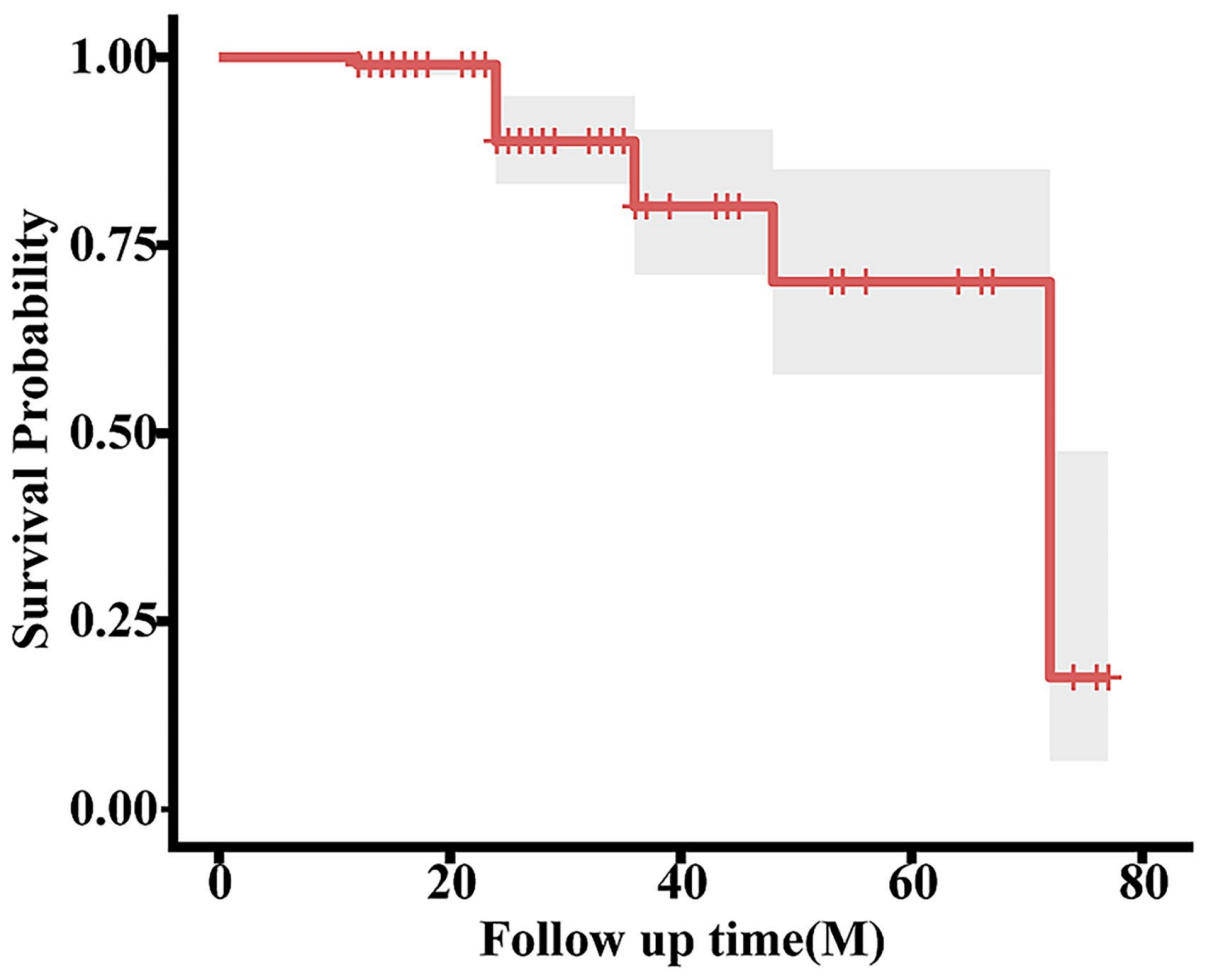
Kaplan–Meier curve demonstrating pterygium recurrence over time. Shaded regions represent the 95% confidence intervals (CIs). The mean follow-up period of included cases was 30.65 ± 16.96 months. The overall recurrence rate was 14.5%. The mean time to recurrence was 42.21 ± 22.13 months.

To further elucidate recurrence patterns, the patient cohort was stratified based on clinical outcomes. Notably, the mean follow-up duration for the non-recurrence group (n = 171) was 27.05 ± 13.16 months, whereas the recurrence group (n = 29) exhibited a significantly longer mean follow-up of 51.83 ± 21.19 months. Within the recurrence cohort, the mean time to recurrence was 42.21 months; this prolonged interval underscores the necessity of extended longitudinal observation to accurately capture late-onset events (Table 2). Furthermore, Kaplan–Meier survival analysis (Figure 1) revealed a median time to recurrence of 72.0 months (95% CI: 64.0–80.0).

**Table 2:** Comparison of population characteristics and clinical factors between non-recurrent and recurrent pterygium group.

Characteristics	Non-recurrent pterygium (*n* = 171)	Recurrent pterygium (*n* = 29)	*p*-value
Gender (Male: Female)	46:125	4:25	0.202
Average age, years	60.27 ± 10.33	67.62 ± 5.79	0.000
Follow-up time, mos	27.05 ± 13.16	51.83 ± 21.19	0.000
Preoperative BCVA (logMAR)	0.57 ± 0.60	0.98 ± 0.75	0.008
Size of pterygium, mm (range)	3.15 ± 0.53	3.07 ± 0.26	0.441
Level of education			0.013
>6 years (%)	58 (66.1)	12 (41.4)	
≤6 years (%)	113 (33.9)	17 (58.6)	
Outdoor Job (%)	134/171 (78.4%)	27/29 (93.1%)	0.110
Sun exposure time, h (range)	4.80 ± 2.17	5.79 ± 1.01	0.000
Surgeon (resident: attending)	111:60	16:13	0.314
Recurrent time, mos	–	42.21 ± 22.13	-
Smoking (%)	8/171 (4.7)	0/29 (0)	0.606
DM (%)	9/171 (5.3)	3/29 (10.3)	0.520
Height (cm)	155.50 ± 8.48	151.76 ± 8.82	0.030
Weight (kg)	56.64 ± 9.80	53.25 ± 7.55	0.039
BMI (kg/m^2^)	23.36 ± 3.23	23.13 ± 2.66	0.718
HDL-C (mmol/L)	1.38 ± 0.31	1.38 ± 0.31	0.990
LDL-C (mmol/L)	3.50 ± 2.66	3.35 ± 0.85	0.755
TG (mmol/L)	1.92 ± 1.70	2.05 ± 1.26	0.690
Total cholesterol (mmol/L)	5.28 ± 1.11	5.27 ± 1.09	0.964

Categorical variables were assessed using a chi-square test and presented as number (%); continuous variables were assessed using a *t*-test and reported as mean ± standard deviation. BCVA, best corrected visual acuity; DM, diabetes mellitus; BMI, body mass index; HDL, high-density lipoprotein; LDL, low-density lipoprotein; TG, triglyceride.

The mean age was significantly higher in the recurrent group (67.62 ± 5.79 years) compared to the non-recurrent group (60.27 ± 10.33 years; *p* < 0.001). Male distribution was 26.90% in the non-recurrent group and 13.79% in the recurrent group (*p* = 0.202). Regarding preoperative BCVA, the recurrent group exhibited a significantly worse mean BCVA (0.98 ± 0.75 logMAR) compared to the non-recurrent group (0.57 ± 0.60 logMAR). The mean size of the initial pterygium did not differ significantly between the non-recurrent (3.15 ± 0.53 mm) and recurrent (3.07 ± 0.26 mm) groups (*p* = 0.441).

Every patient underwent pterygium excision combined with limbal conjunctival autograft. In the non-recurrent group, 111/171 (64.91%) procedures were performed by residents and 60/171 (35.09%) by senior attending physicians; in the recurrent group, these proportions were 16/29 (55.17%) and 13/29 (44.83%), respectively (*p* = 0.314).

The smoking rate in the non-recurrent group (4.7%) was higher than in the recurrent group (0%), though this was not statistically significant (*p* = 0.606). Education levels were significantly higher in the non-recurrent group, where 66.1% of patients had reached higher education levels compared to 41.4% in the recurrent group (*p* = 0.013). No significant differences were found between groups regarding anthropometric parameters, such as BMI or lipid profiles. A detailed comparison of clinical factors is presented in Table 2.

To identify independent predictors of pterygium recurrence, we performed both univariate and multivariate logistic regression analyses. In the univariate analysis, several variables were found to be significantly associated with a higher risk of recurrence (all *p* ≤ 0.05). These included: advanced age at onset (OR = 1.061; 95% CI: 1.021–1.103; *p* = 0.003), poorer preoperative BCVA (OR = 2.332; 95% CI: 1.358–4.004; *p* = 0.002), lower educational attainment (OR = 0.362; 95% CI: 0.162–0.810; *p* = 0.013), increased sun exposure (OR = 1.477; 95% CI: 1.027–2.126; *p* = 0.036), and shorter stature (OR = 0.950; 95% CI: 0.907–0.996; *p* = 0.033).

Subsequently, all variables that demonstrated statistical significance in the univariate analysis were included in a multivariate logistic regression model to adjust for potential confounding effects. As detailed in Table 3, the multivariate analysis identified age as the only independent risk factor for recurrence in this cohort. Specifically, for every one-year increase in age at onset, the risk of recurrence rose significantly, with an adjusted OR of 5.203 (95% CI: 1.565–17.305; *p* = 0.007). Conversely, other factors—including preoperative BCVA (*p* = 0.474), level of education (*p* = 0.397), sun exposure time (*p* = 0.297), and height (*p* = 0.707), did not maintain statistical significance in the final multivariate model.

**Table 3:** Univariate and multivariate analysis of risk factors for pterygium recurrence after limbal-conjunctival autografting.

Variable	Univariate analysis		Multivariate analysis	
	*p*-value	OR (95% CI)	*p*-value	OR (95% CI)
Age	0.003	1.061 (1.021–1.103)	0.007	5.203 (1.565–17.305)
Preoperative BCVA	0.002	2.332 (1.358–4.004)	0.474	1.266 (0.663–2.420)
Level of education	0.013	0.362 (0.162–0.810)	0.397	0.664 (0.257–1.713)
Sun exposure time	0.036	1.477 (1.027–2.126)	0.297	1.233 (0.831–1.829)
Height	0.033	0.950 (0.907–0.996)	0.707	0.989 (0.934–1.047)

BCVA, best corrected visual acuity.

## 4 Discussion

This study delineates the demographic and clinical profiles of patients with non-recurrent and recurrent pterygium in southern remote regions of China, elucidating specific risk factors associated with recurrence. Our analysis revealed significant divergences between the non-recurrent and recurrent cohorts, particularly regarding age, height, preoperative BCVA, educational attainment, and cumulative sunlight exposure. In multivariate analysis, advanced age at onset was identified as a statistically significant risk factor for recurrence. These findings provide critical insights into the etiology of recurrence within this specific demographic and establish a foundation for tailored clinical management.

The recurrence rate in our cohort was 14.5%, with a mean time to recurrence of 42.21 ± 22.13 months. While comparable to the 17% observed in Saudi Arabia, this rate notably exceeds the 7.65% reported in urban Chinese populations and the 11.4% observed in the United States (11–13). Contrary to previous literature positing a postoperative recurrence latency of 3–6 months (3, 14, 15), our data demonstrates a substantially prolonged mean recurrence interval of approximately 3.5 years, aligning more closely with the 34.2 months reported by Aidenloo et al. (16). We hypothesize that this extended latency is attributable to the advanced age of our study population. Given that younger subjects typically exhibit more aggressive epithelialization, collagen synthesis, and inflammatory responses, it is biologically plausible that the physiological processes driving recurrence manifest more indolently in older individuals (3).

With respect to environmental factors, the recurrence group experienced significantly longer durations of sunlight exposure than the non-recurrent group. This observation aligns with established literature implicating ultraviolet radiation as a critical factor in both pterygium pathogenesis and postoperative recurrence (17, 18). Mechanistically, we attribute this association to inflammation; Chen et al. observed that postoperative inflammatory responses are exacerbated in individuals with extensive sun exposure history (12). Ultraviolet radiation is known to potentiate recurrence risk by promoting fibrovascular proliferation and inducing pro-inflammatory cytokines (e.g., IL-6, IL-8, TNF-*α*) in the limbal region (19). Evidence suggests that the fibrovascular tissue in recurrent pterygium is notably more severe; however, there is a paucity of data regarding the mechanisms driving this recurrence (17, 20). This highlights a critical need to further investigate the underlying pathophysiology of the disease.

Socioeconomic determinants also appear to exert a pivotal influence. The recurrence group demonstrated lower educational attainment compared to the non-recurrent group, a finding paralleling Hashemi et al. (21). We posit that educational attainment functions as a surrogate marker for lifestyle and health literacy. Individuals with higher education typically achieve higher socioeconomic status, correlating with reduced occupational outdoor exposure. Furthermore, education likely mitigates recurrence rates by enhancing health awareness, facilitating access to medical resources, and improving adherence to postoperative care and ultraviolet protection measures.

We further observed an inverse correlation between preoperative BCVA and recurrence. While no significant difference in pterygium size was detected between groups, compromised preoperative vision may indicate underlying ocular surface pathologies, such as severe dryness or inflammation, which are known to predispose patients to recurrence (3, 11) However, interpreting BCVA in this elderly cohort warrants caution. Although pterygium-induced astigmatism compromises visual acuity, age-related comorbidities such as cataracts act as significant confounders (22–24). Consequently, future research must delineate the specific impact of preoperative visual function on postoperative outcomes.

A salient feature of our data is the significantly advanced age of the recurrent cohort compared to the non-recurrent group, a factor that retained significance in multivariate analysis. This trend stands in marked contrast to the prevailing consensus identifying younger age as a principal risk factor; notably, Aidenloo et al. (16) reported a 3.5-fold increased risk in patients under 45 (3, 14, 15). We ascribe this discrepancy to the unique demographic landscape of remote China, which is characterized by a predominance of elderly women. In this setting, older adults frequently engage in prolonged agricultural labor involving intense ultraviolet exposure. Furthermore, this population often exhibits delayed health-seeking behaviors and suboptimal adherence to postoperative regimens. Our findings suggest that in southern rural China, environmental stressors and behavioral patterns supersede the biological protective effects typically associated with advanced age.

Consistent with previous studies, we observed no significant association between recurrence and systemic factors such as BMI, lipid profiles, or smoking status (11, 12, 25). While this suggests the predominance of local ocular etiology, the potential influence of systemic health on pterygium recurrence merits further exploration in larger prospective cohorts.

Given these findings, optimizing surgical intervention is paramount. While we utilized the standard limbal-conjunctival autograft, the 14.5% recurrence rate suggests that traditional approaches may be insufficient for high-risk rural populations. Emerging evidence underscores the meticulous management of Tenon’s capsule as a critical determinant of surgical success (26–28). The Tenon’s tissue adjacent to the graft may act as a potential reservoir for fibroblast proliferation, thereby contributing to pterygium recurrence. Consistently, Ciftci et al. demonstrated that extensive Tenon’s layer excision combined with limbal-conjunctival autograft significantly mitigates recurrence risks compared to isolated autografts—a technique that also offers a shorter learning curve for surgeons (29, 30). Given the specific risk profile of our study population, the integration of Tenon’s layer excision with limbal-conjunctival autograft may represent a superior therapeutic strategy to counteract the elevated recurrence rates identified in this investigation.

Several limitations to this study warrant consideration. First, the retrospective nature of the data inherently limits causal inference; thus, the observed associations necessitate confirmation through longitudinal designs. Second, the absence of detailed morphological classification—specifically regarding location and severity grading—restricts the generalizability of our findings to specific pterygium subtypes. Third, our assessment of environmental risk was confined to the duration of outdoor exposure and did not account for other irritants such as wind, sand, or specific ultraviolet indices. Finally, the analysis of visual function was constrained by the lack of refractive data (e.g., corneal astigmatism) and the inability to fully adjust for cataracts as a confounding factor. Despite these limitations, we are currently undertaking prospective investigations incorporating these parameters to deepen our understanding of pterygium pathogenesis and explore new therapeutic avenues.

## 5 Conclusion

In summary, this study establishes that the recurrence rate of pterygium in remote areas of China is 14.5%, with a relatively prolonged mean recurrence interval of 42.21 months. Patients experiencing recurrence in these regions are characterized by advanced age, poorer preoperative BCVA, lower educational attainment, and greater cumulative sun exposure. Moreover, our multivariate analysis confirms that older age at onset is a significant risk factor for recurrence in this specific population. Consequently, clinical management in these regions should prioritize elderly patients through extended follow-up periods, enhanced medical support, and rigorous health education regarding postoperative care.

## Data Availability

The raw data supporting the conclusions of this article will be made available by the authors, without undue reservation.
